# Associations of nutritional awareness, body mass index, mental health, and fitness components among undergraduate university students

**DOI:** 10.3389/fnut.2025.1614296

**Published:** 2025-09-24

**Authors:** Laila Fathi Zaid AlKilani, Salwa Saad Awad, Jozaa Zaidan ALTamimi, Fatimah Sayer Alharbi

**Affiliations:** ^1^Department of Physical Sport Sciences, College of Sport Sciences and Physical Activity, Princess Nourah bint Abdulrahman University, Riyadh, Saudi Arabia; ^2^Department of Rehabilitation Sciences, College of Health and Rehabilitation Sciences, Princess Nourah bint Abdulrahman University (PNU), Riyadh, Saudi Arabia; ^3^Department of Sports Health, College of Sport Sciences and Physical Activities, Princess Nourah bint Abdulrahman University, Riyadh, Saudi Arabia; ^4^Department of Mental Health Psychology, College of Education and Human Development, Princess Nourah bint Abdulrahman University (PNU), Riyadh, Saudi Arabia

**Keywords:** physical activity, mental health, weight, female, university students

## Abstract

**Introduction:**

The study generally aimed to examine nutritional awareness, body mass index, mental health, dietary awareness, and components of physical fitness among university students by investigating the health behaviors, physical activity, diet, sleep, internet use, and mental health of 153 undergraduate university students.

**Methods:**

This study employed a cross-sectional quantitative survey method to evaluate the risky and health-related behaviors, psychological health, and health-related behaviors of female university students.

**Results:**

Among them, 66% did not engage in high-intensity physical activities, and 54.9% did not participate in moderate-intensity workouts. Of the participants, 52.9% ate two meals a day, and 43.1% only sometimes ate breakfast. According to the survey, 56% recognized smoking as a contributing factor to health problems, and 60% said stress had a major effect on health problems. Moreover, the results reveal a statistically significant relationship between weight category and depression symptoms (*r* = −0.06, *p =* 0.53) and between physical activity and depression symptoms (*r =* −0.11, *p =* 0.23).

**Discussion:**

The findings call which calls for encouraging the increase of such studies in the future and conducting them on a larger sample and encouraging the adoption of university programs aimed at raising awareness of the importance of physical activity, healthy nutrition, and mental health.

## 1 Introduction

University students must overcome a number of obstacles throughout their formative years in college, including adjusting to new schedules, building social networks, and adapting to changes in their physical and social environments (1). Students at college are more likely to engage in risky health behaviors in this period of their lives, including stress, suboptimal dietary habits, and physical inactivity, all of which are known to negatively influence well-being (2, 3). A number of studies conducted in Saudi Arabia indicate that college students have bad habits related to their nutrition and lifestyle (4–7). Additional research conducted on Saudi health college students found that, despite being aware of the need to adopt healthy behaviors, they did not adhere to the predetermined criteria in their entirety (8–11). As a result of these behavioral features, Saudi college students are more likely to experience weight gain, which, in turn, makes them more susceptible to illness (12, 13).

Health behaviors are actions that impact the health of an individual or a group, including food choices and physical activity (PA). The bases of health behaviors include sites of housing, work, educational opportunities, and recreational pursuits. Diabetes, cancer, heart disease, and stroke are the major causes of death across the globe, according to the World Health Organization (14), which recognizes these conditions specifically as chronic diseases, and behavioral risk factors correlate considerably with the incidence and mortality of chronic diseases in adults (15). These risk factors include obesity, sedentary behavior, and physical inactivity. The risk of non-communicable illnesses is increased when individuals make lifestyle choices that are unhealthy and have an elevated body weight, and students at higher education institutions are more likely to engage in unhealthy habits and experience obesity (15). Gaining weight, particularly when it results in being overweight or obese, leads to cardiovascular and mental health (MH) illness (16).

The influence of peers and the availability and affordability of food are examples of sociocultural and environmental factors that may impact behavior. Students believe that their nutritional knowledge (NK) and perspectives on the health advantages of food, as well as their personal attributes, such as their ability to cook, can influence their behavioral patterns surrounding food consumption (17). Research conducted on college students indicates a favorable correlation between nutritional awareness and greater consumption of fruits, dairy products, protein, and whole grain meals among them, as well as other dietary behaviors, such as reading product labels (18).

Previous research on college students that took a cross-sectional approach revealed a lack of knowledge on a variety of dietary themes. The students gave wrong responses to more than 50% of the questions concerning fruits and vegetables and milk and their substitutes, as well as fermented dairy products (19), vitamin D (20, 21), food labeling (22), and the connection between nutrition and chronic illnesses (23–25). According to a growing body of research, behavioral risk factors, which include physical inactivity, obesity, and sedentary behavior, are found to co-occur in children and to increase the risk of chronic illnesses beyond the cumulative effects of each component (25, 26).

At Dammam University in Saudi Arabia, first-year students studying health sciences were the subjects of a study (27) that investigated the influence of the duration of weekly training, the amount of time spent sitting each day, and body mass index (BMI) on exercise-induced heart rate and systolic blood pressure. According to the findings, more than 65% of the participants did not achieve the amount of PA advised for health advantages. Moreover, research indicates that television viewing, internet use, and academic pressures collectively contribute to a decline in interest in physical activity (28). Inactivity and a sedentary lifestyle are two major factors that contribute to poor health (29, 30). The concept of risk reduction emphasizes the necessity of young people engaging in a basic amount of daily exercise as opposed to maintaining a conspicuously sedentary lifestyle because while many sedentary activities may pose no danger when done in moderation, full inactivity can be deleterious.

A correlation exists between PA and improved MH. Enhanced PA is linked to mental and social well-being (31–37). A higher level of PA was found to correlate positively with overall well-being, whereas it has a negative correlation with symptoms of anxiety and depression (38).

The quality of life of university students who engage in regular PA is positively correlated with PA, whereas levels of stress, anxiety, and depression are negatively correlated with PA (39).

The rapid changes in a student’s biological, emotional, cognitive, and social development immediately following their entry into high school or university affect the student’s conduct (40). During this time, adolescents and young adults typically exhibit a sense of curiosity and participate in a wide variety of activities that are necessary for growth. When it comes to some activities that are deemed undesirable and damaging to both the individual and society; however, several civilizations have established boundaries on those actions (40).

Understanding behavior and health (risk awareness) is essential to adopt good lifestyle choices. Risk awareness is linked to beliefs and attitudes, and multiple studies (41, 42) show that health practices are implemented based on their perceived benefits. People change their habits based on science, medicine, and evolving social consensus (41). Healthy habits include a low-fat diet, regular exercise, and fruit and vegetable consumption. Early childhood and adolescent health habits can impact subsequent disease (42). PA, diet, and regular health exams are significantly linked to risk factors, even though many illnesses’ causes are unknown.

Focusing on university students is essential to establish a robust, consistently updated database concerning their health and well-being, which is necessary for developing targeted and sustainable health promotion interventions and strategies, as universities increasingly face pressure to foster a healthy academic environment with constrained resources. This study examined the correlation between PA and elevated MH among undergraduates. It specifically sought to ascertain whether these associations differ according to the intensity of PA (low, medium, or high) and the students’ NK.

## 2 Materials and methods

### 2.1 Study design and participants

This study employed a cross-sectional quantitative survey method to evaluate the risky and health-related behaviors, psychological health, and health-related behaviors of female university students. Restricting the sample to women helps reduce variability that may arise from gender-related differences in experiences, behaviors, and perceptions of the study variables. In addition, female students constitute a substantial proportion of the student population, yet their perspectives have often been underrepresented in previous research. Given the growing focus on women’s health and the unique challenges they face, the researchers chose to initiate the program with female participants. Once the program demonstrates success, it will be expanded to include both male and female students in future research. The study was carried out during the 2023 academic year. The students completed a questionnaire with the following parts:

Part 1: Sociodemographic data
Part 2: Physical activity
Part 3: Tobacco and drug use
Part 4: Frightening experience in life
Part 5: Center for Epidemiologic Studies Depression Scale Revised (CESD-R10)
Part 6: Eating habits
Part 7: Sleep
Part 8: Other behaviors
Part 9: Internet usage
Part 10: General life situation
Part 11: Concerns about knowledge of various health problems
Part 12: Social
Part 13: Happiness
Part 14: Physical measurements

Nutritional awareness is being aware of the nutritional value of different foods, understanding how to make informed food choices, and recognizing the impact of diet on overall health and well-being. The several elements of physical fitness required for general health and well-being are referred to as fitness components. Usually, these elements consist of cardiovascular endurance, muscular strength, range of motion in muscles and joints, and body composition. One may evaluate several facets of body composition using BMI as follows:

- BMI < 18.5: possibly unhealthily low body weight (underweight)
- BMI = 18.5–24.9: normal weight
- BMI = 25–29.9: possibly harmful high body weight (overweight)
- BMI ≥ 30: high risk of health issues connected to excess weight (obesity)

Using BMI, scientists can investigate links between dietary knowledge, MH, exercise components, and other facets of body composition, not only weight increase.

The survey instrument was developed to assess students’ perspectives on healthy habits and physical activities that contribute to their physical and mental well-being. The participants received explanations of the study, the health-related activities, and the purpose of the survey. Trained student researchers verified whether the students in the sample had any questions and understood the questionnaire before the participants submitted their responses. One department was randomly chosen from each college in the university, with the probability of selection correlated to department size.

#### 2.1.1 Inclusion criteria

A total of 153 students aged 18–25 years from all colleges who did not have a history of psychological issues or a prior diagnosis of physical disability met the study’s inclusion criteria. About 170 responses were collected, 13 of which were excluded due to inaccurate data provided by the respondents.

#### 2.1.2 Exclusion criteria

- Students with severe medical conditions that could impact their nutritional awareness, BMI, MH, or fitness components (e.g., diabetes, heart disease, and severe MH conditions).
- Students who were competitive athletes or fitness professionals, as their nutritional awareness, BMI, MH, and fitness components could differ from those of non-athletes/non-fitness professionals.
- Students with disabilities that could impact their ability to participate in PA or fitness components (e.g., mobility impairments and chronic pain).

### 2.2 Study instruments

Saudi college students were approached *via* SurveyMonkey online. The questionnaire was designed with three main sections. The initial subdivision focused on the respondents’ sociodemographic and general health information, including questions related to age, sex, educational background, and health status. The second and third sections consisted of self-report questionnaires to assess their engagement in PA and health-related behaviors as well as MH. These inquiries were carefully chosen to provide deep insights into the participants’ knowledge concerning their PA, general health, and MH.

Each section of the questionnaire was explained and its purpose clarified to avoid any confusion among the participants. A pilot study was conducted before conducting the study on 30 volunteer participants to evaluate the questionnaire, and validity and reliability measures were conducted.

#### 2.2.1 Sociodemographic and general information

In the sociodemographic questionnaire, participants were asked to provide their age, rate their health status as excellent, very good, good, or poor, and indicate their marital status, residence status, previous semester’s Grade Point Average (GPA), and perception of their body weight.

#### 2.2.2 International Physical Activity Questionnaire

The Short International Physical Activity Questionnaire (IPAQ) explored the extent to which students engaged in PA while at college, during commutes, during leisure time, and in other activities that are considered forms of physical exercise. The number of days per week that were dedicated to moderate activities was documented in the self-administered questionnaire, which offers standard tools to gather data on PA related to health that are comparable across borders (43). Participants were informed that they could use smartphone or smartwatch apps to remember their level and duration of physical activity.

#### 2.2.3 Health Belief Model

The participants were asked to rate the importance of a series of behaviors for health maintenance according to their importance to health. The Health Belief Model (HBM) (44) is a psychological framework that focuses on people’s attitudes and beliefs to explain and forecast health-related behaviors.

#### 2.2.4 Health Risk Appraisal

The risk-knowledge items of the Health Risk Appraisal (HRA) ask participants to indicate whether each of dietary fat, exercise, smoking, and alcohol consumption contributes to five different health problems (heart disease, lung cancer, mental illness, breast cancer, and high blood pressure) (45, 46). It describes a person’s chances of becoming ill or dying from selected diseases. HRA has become a popular approach to help people identify the risks associated with personal characteristics (biological, lifestyle, and family history).

#### 2.2.5 The CESD-R10

The CESD-R10 assessment evaluates depression symptoms over the previous week using 10 questions, each scored from 0 (rarely) to 3 (frequently). The range of scores is 0–30, with higher scores representing a more depressed mood. A score of 10 is commonly used as a cut-off point for potential depression. This cut-off suggests either a significant number of persistent symptoms or a broader range of symptoms for shorter periods. The survey is designed for ease of use and typically takes 2–5 min (47).

### 2.3 Data collection procedure

Trained researchers measured the participants’ physical characteristics with standardized measures. The standing height of each student was measured to the nearest 0.1 cm without shoes using a stature meter. The participants were weighed to the nearest 0.01 kg on a load cell–operated digital scale with a weighing capacity of 140 kg. The scale was first calibrated with a standard weight and was checked daily. BMI was computed for each participant by dividing weight in kilograms by the square of height in meters. Waist circumference was measured at a point one-third the distance from the hips to the shoulders, using a non-stretchable tape measure (42). Hip circumference was measured around the hips at a point 4 cm below the superior anterior iliac spine (48, 49). The waist-to-hip ratio was calculated for each participant by dividing the waist measurement by the hip measurement.

### 2.4 Ethical considerations

The Institutional Review Board (IRB) of Princess Nourah bint Abdulrahman University, Riyadh, Saudi Arabia, reviewed and approved the study (approval no. 21-0429). The participants were given a description of the study and informed of their information’s confidentiality at the outset of the survey, and they provided informed consent.

### 2.5 Data analysis

The acquired data were analyzed using descriptive statistics; frequency and percentage were used for the quantitative variables. The relationship between the respondents’ level of PA, health behavior, and personal characteristics were examined using the Chi-squared test. The collected data were arranged in an Excel sheet as a database file to be analyzed using the Statistical Package for the Social Sciences (SPSS), which was also utilized to measure the distribution of frequencies of the variables under consideration. The Chi-squared test was used to analyze the association between variables.

## 3 Results

### 3.1 Demographic data

**Figure 1:**
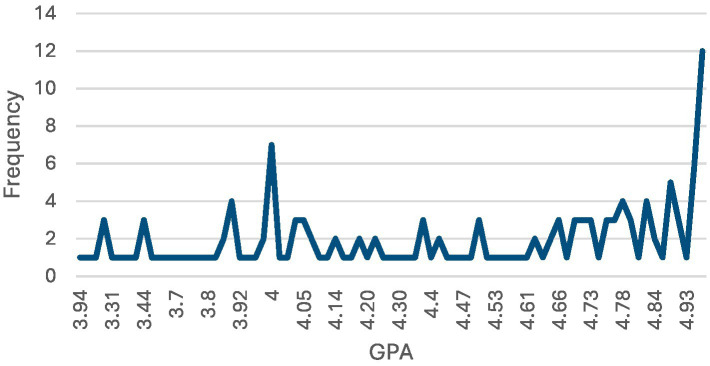
Line graph showing GPA distribution.

**Figure 2:**
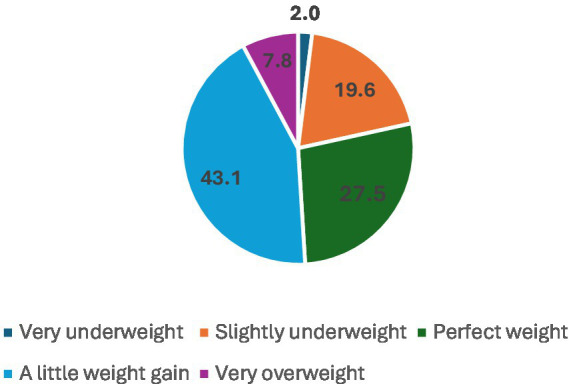
Weight categories.

The participant demographics indicate that the vast majority of respondents, 98.0% (*n =* 150), spoke Arabic, with only 2.0% (*n =* 3) speaking English. In terms of age, the largest group was 18-year-olds, making up 49.0% (*n =* 75) of the participants, followed by 19-year-olds at 41.2% (*n =* 63). Most participants, 92.8% (*n =* 142), resided with their parents, but a small portion, 7.2% (*n =* 11), lived in university housing. Regarding health conditions, most respondents rated their health as very good, accounting for 41.8% (*n =* 64), followed by those who rated it as good at 24.2% (*n =* 37) (see Table 1). All the students who participated were unmarried during their foundation year at the university. Figure 1 shows their GPA distribution, which ranges from 0 to 5. The participants’ responses regarding how they perceived their body weight are given in Figure 2.

**Table 1:** Demographic data.

Demographic data		*N*	%
Language spoken	Arabic	150	98.0
	English	3	2.0
Age	18	75	49.0
	19	63	41.2
	20	14	9.2
	21	1	0.7
Residence	University housing	11	7.2
	With parents	142	92.8
Health condition	Excellent	36	23.5
	Good	37	24.2
	Moderate	13	8.5
	Poor	3	2.0
	Very good	64	41.8
	Total	153	100.0

### 3.2 Physical activity

**Figure 3:**
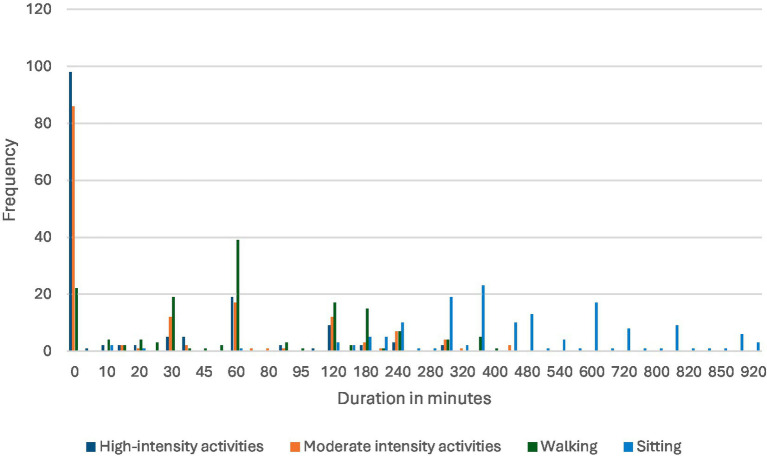
Frequency of different activity intensities over varying durations.

The participants’ data reveal that 66.0% (*n =* 101) did not engage in any high-intensity PA. Additionally, 54.9% (*n =* 84) did not participate in moderate-intensity workouts. Regarding walking, 34.0% (*n =* 52) of the participants walked every day, with 25.5% (*n =* 39) spending 60 min walking daily. Regarding sedentary behavior, 15.0% (*n =* 23) of the participants sat for 360 min (6 h) daily. Figure 3 shows the time spent on high-intensity and low-intensity activities, walking, and sedentary activity.

### 3.3 Life experiences

The participant responses indicate that 54.2% (*n =* 83) had avoided being reminded of a frightening or upsetting experience by staying away from certain places, people, or activities. Additionally, 66.0% (*n =* 101) had lost interest in activities that were once important or enjoyable. A significant portion, 63.4% (*n =* 97), had begun to feel more isolated or distant from other people, and 40.5% (*n =* 62) found it hard to feel love or affection for other people. Only 17.6% (*n =* 27) had begun to feel that there was no point in planning for the future. Furthermore, 65.4% (*n =* 100) had more trouble than usual falling asleep or staying asleep, and 66.7% (*n =* 102) became jumpy or easily startled by ordinary noises or movements.

### 3.4 CESD-R10

The largest responses for each statement regarding feelings during the past week reveal that 36.6% (*n =* 56) were rarely bothered by things that usually did not bother them, whereas 34.6% (*n =* 53) often had trouble keeping their mind on what they were doing. Similarly, 34.6% (*n =* 53) often felt that everything they did required an effort. Depression was rarely felt by 29.4% (*n =* 45) of the participants. Hopefulness about the future was felt a little (a day or two) by 37.9% (*n =* 58). Fearfulness was often experienced by 27.5% (*n =* 42), and frequent restless sleep was reported by 28.1% (*n =* 43). Happiness was felt equally a little and often by 37.9% (*n =* 58) of the participants. Loneliness was rarely felt by 31.4% (*n =* 48), and 43.8% (*n =* 67) rarely found it difficult to get going. Scores were assigned from 1 to 4 for rarely to most days, respectively. The scores were reversed for items 5 and 8. Table 2 gives the mean (*M*), standard deviation (*SD*), and complete responses.

**Table 2:** CESD responses.

For each of these statements, please indicate how often you felt this way during the past week.	Rarely (less than a day)		A little (a day or two)		Often (3–4 days)		Most (5–7 days)		Mean	*SD*
	*n*	%	*n*	%	*n*	%	*n*	%		
I was bothered by things that usually do not bother me.	56	36.6	49	32	36	23.5	12	7.8	2.0	1.0
I had trouble keeping my mind on what I was doing.	19	12.4	51	33.3	53	34.6	30	19.6	2.6	0.9
I felt that everything I did was an effort.	29	19.0	36	23.5	53	34.6	35	22.9	2.6	1.0
I felt depressed.	45	29.4	42	27.5	30	19.6	36	23.5	2.4	1.1
I felt hopeful about the future.	18	11.8	58	37.9	50	32.7	27	17.6	2.4	0.9
I felt fearful.	39	25.5	41	26.8	42	27.5	31	20.3	2.4	1.1
My sleep was restless.	28	18.3	42	27.5	43	28.1	40	26.1	2.6	1.1
I was happy.	19	12.4	58	37.9	58	37.9	18	11.8	2.5	0.9
I felt lonely.	48	31.4	36	23.5	30	19.6	39	25.5	2.4	1.2
I could not get going.	67	43.8	38	24.8	22	14.4	26	17.0	2.1	1.1

### 3.5 Nutrition

**Figure 4:**
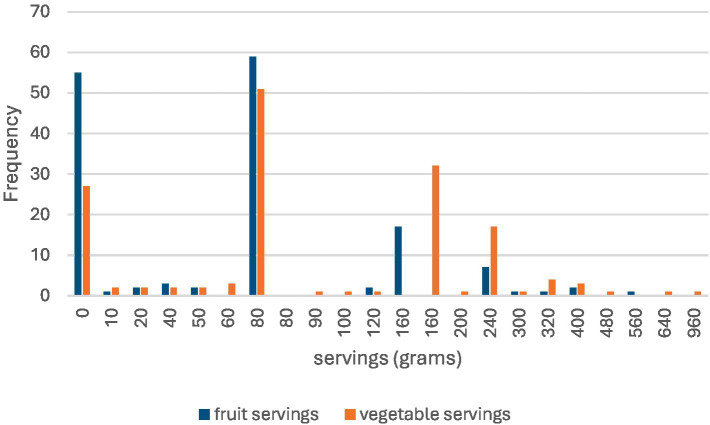
Fruit and vegetable consumption.

The responses indicate that breakfast was consumed occasionally by 35.3% (*n =* 54) of the participants, with 34.6% (*n =* 53) eating it almost every day and 30.1% (*n =* 46) rarely having it. Most participants, 52.9% (*n =* 81), had two meals a day, whereas 24.2% (*n =* 37) had three meals a day. Regarding between-meal snacks, 35.3% (*n =* 54) had two snacks a day, followed by 28.8% (*n =* 44) who had one snack a day. Meals with meat were consumed seven times a week by 31.4% (*n =* 48) of the participants, and 30.1% (*n =* 46) consumed them either once or three times a week. Finally, 43.1% (*n =* 66) usually added salt to their meals, and 28.8% (*n =* 44) added it occasionally. Figure 4 gives the fruit and vegetable servings consumed by the participants in the past 7 days.

### 3.6 Food consumption

The responses indicate that 24.8% (*n =* 38) of the participants have consumed chocolate or candy three to six times in the past 7 days. Sugared tea or coffee was consumed once daily by 25.5% (*n =* 39) of participants. Regarding soft drinks, 48.4% (*n =* 74) had drunk them less than once per day in the past 30 days. The frequency of fast-food consumption in the past 30 days was highest for once, with 22.9% (*n =* 35) of participants reporting that frequency.

Table 3 shows the complete response. Scores were assigned for consumption frequency, ranging from 0 (never) to 6/7 (more than once a day), with options 0 (never) and 7 (every day). A cumulative score of unhealthy food habits was calculated, with a least possible score of 0 and a highest possible score of 26. The lowest actual score of 1 was obtained by 1.3% (*n =* 2), and the highest actual score of 22 was obtained by 0.7% (*n =* 1).

**Table 3:** Food consumption.

Food	Consumption pattern	N	%
Chocolate or candy in the past 7 days	Never	4	2.6
	Rarely	16	10.5
	1–2 times/week	30	19.6
	3–6 times/ week	38	24.8
	Once daily	32	20.9
	More than 1 time/day	33	21.5
Sugared tea or coffee in the past 7 days	Never	28	18.3
	Rarely	32	20.9
	1–2 times/week	23	15.0
	3–6 times/week	17	11.1
	Once daily	39	25.5
	More than 1 time/day	14	9.1
Frequency of soft drinks in the past 30 days	Never in past 30 days	41	26.8
	Less than 1 time/day	74	48.4
	Once daily	22	14.4
	Twice/day	9	5.9
	3 times/day	2	1.3
	4 times/day	2	1.3
	5 times or more/day	3	2.0
Frequency of fast food in the past 30 days	0	27	17.6
	1	35	22.9
	2	25	16.3
	3	23	15.0
	4	14	9.2
	5	9	5.9
	6	6	3.9
	7	14	9.2

**Figure 5:**
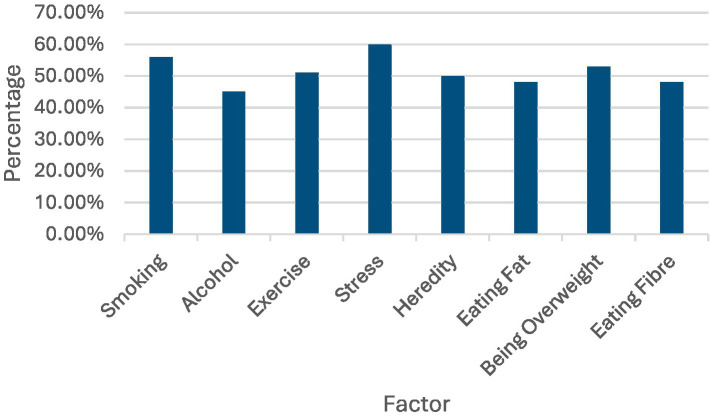
Factors percentage values for factors affecting health.

The participants’ perceptions of various factors influencing health problems were analyzed. Stress emerged as the most recognized factor, with 60% of participants identifying it as a major influence on health issues, such as heart disease, lung cancer, mental illness, breast cancer, and high blood pressure. Smoking was also prominently identified, with 56% of participants recognizing its impact across the same health conditions. Being overweight was recognized by 53% of the respondents, highlighting its perceived role in contributing to multiple health problems. Exercise was identified by 51% of participants, indicating an awareness of its importance in maintaining health. Heredity, with 50% recognition, signifies the acknowledgment of genetic predispositions in health. Both eating fat and fiber were identified by 48% of participants, reflecting an understanding of their dietary impacts on health. Alcohol was noted by 45% of respondents as a contributing factor (see Figure 5).

### 3.7 Sleep

The data reveal that the participants’ most common duration of sleep was 8 h, reported by 20.3% (*n =* 31) of respondents. This is followed by 6 h, reported by 17.6% (*n =* 27), and 7 h, reported by 15% (*n =* 23). In terms of sleep problems, 30.7% (*n =* 47) reported having moderate sleep problems, whereas 27.5% (*n =* 42) reported both light and intense sleep problems. Only 14.4% (*n =* 22) reported having no sleep problems.

### 3.8 Internet usage

**Figure 6:**
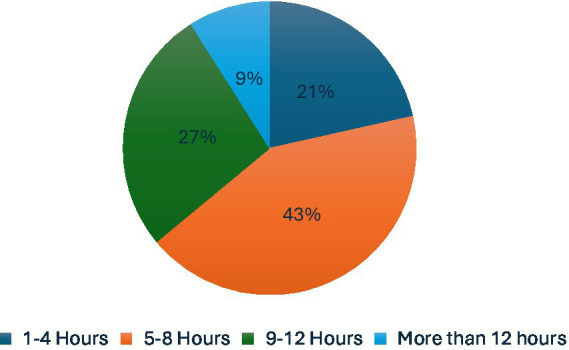
Time spent on Smart devices or cell phones.

**Figure 7:**
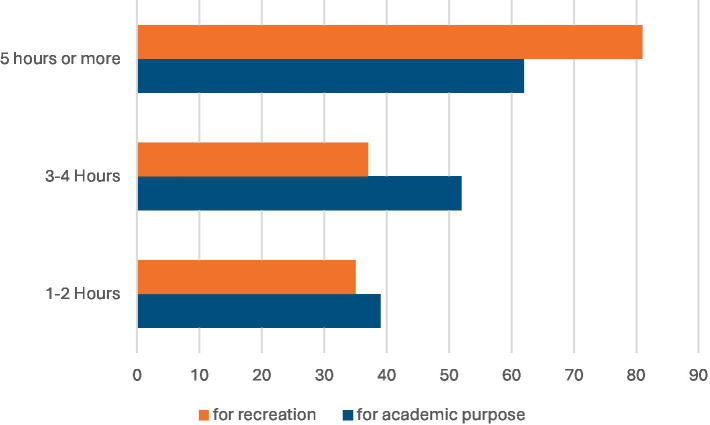
Time spent on the INTERNET for academic or recreational purposes.

A total of 43% of participants reported spending 5–8 h per day using the internet, whether on smart devices or mobile phones, while 27% of participants reported spending 1–4 h per day, and 21% of participants reported spending 9–12 h per day online (Figure 6). Approximately 62 participants (40.5%) spent 5 h or more online for academic purposes, and 81 participants (52.9%) for entertainment purposes. Additionally, 52 participants (34%) reported spending 3–4 h per day online for academic purposes, and 37 participants (24.2%) for entertainment purposes (Figure 7).

A majority of participants, 68.0% (*n =* 104), felt preoccupied with the internet and/or smartphone. A smaller portion, 47.7% (*n =* 73), felt the need to use the internet and/or smartphone with increasing amounts of time to achieve satisfaction. About 52.3% (*n =* 80) had repeatedly made unsuccessful efforts to control, cut back, or stop internet and/or smartphone use. Similarly, 49.0% (*n =* 75) felt restless, moody, depressed, or irritable when attempting to cut down or stop internet and/or smartphone use. A significant majority, 69.3% (*n =* 106), stayed online longer than originally intended. Only 26.1% (*n =* 40) had jeopardized or risked the loss of significant relationships, jobs, or educational or career opportunities because of internet and/or smartphone use. Additionally, 26.8% (*n =* 41) had lied to family members, therapists, or others to conceal the extent of their involvement with the internet and/or smartphones. Finally, 76.5% (*n =* 117) used the internet and/or smartphones as a way of escaping from problems or relieving a dysphoric mood.

### 3.9 Life

Responses for the seven items were collected on a 7-point Likert scale ranging from 1 (strongly disagree) to 7 (strongly agree). The results indicate varying tendencies among the participants regarding their perceptions of control and helplessness in their lives. On average, the participants disagreed with the statement “There is little I can do to change many of the important things in my life” (*M* = 2.72, *SD* = 1.172), suggesting a moderate sense of control. Participants also tended to agree that they felt helpless in dealing with problems (*M* = 3.1, *SD* = 1.236). There was moderate agreement with the notion that achieving desired outcomes was in their own hands (*M* = 3.63, *SD* = 1.18), and a stronger agreement that future outcomes depended on themselves (*M* = 4.01, *SD* = 1.064). Conversely, there was a slight disagreement with having little control over things that happened to them (*M* = 2.67, *SD* = 1.18). Finally, the participants tended to moderately agree that they could accomplish anything they set their mind to (*M* = 3.35, *SD* = 1.155).

The responses reveal that the largest percentage of participants, 43.1% (*n =* 66), completely disagreed with the statement “If I were sick and needed someone to take me to a doctor, I would have trouble finding someone.” Similarly, only 32.0% (*n =* 49) of participants responded “Correct” to the statement “I feel that there is no one I can share my most private concerns with.” Finally, the largest response for the statement “I feel a strong emotional bond with at least one other person” was “Correct,” with 29.4% (*n =* 45) of participants. The complete responses are given in Table 4.

**Table 4:** Support.

Support scale	Completely wrong		Wrong		Correct		Totally correct	
	*n*	%	*n*	%	*n*	%	*n*	%
If I were sick and needed someone to take me to a doctor, I would have trouble finding someone.	66	43.1	48	31.4	22	14.4	17	11.1
I feel that there is no one I can share my most private concerns with.	29	19	40	26.1	49	32	35	22.9
I feel a strong emotional bond with at least one other person.	21	13.7	44	28.8	43	28.1	45	29.4

To assess the participants’ perception of their happiness, data for four items were gathered on a 7-point Likert scale. Scores were assigned from 1, “not a very happy person,” to 7, “a very happy person.” The results indicate that the participants generally considered themselves to be happy, with a mean score of 3.67 (*SD* = 1.701) for the statement “In general, I consider myself a very happy person.” Compared to their peers, the participants also tended to view themselves as happier, with a mean score of 3.54 (*SD* = 1.743). When asked the extent to which they identified with the characterization of a person who was generally very happy and enjoyed life regardless of circumstances, the participants had a mean score of 3.46 (*SD* = 1.777). Conversely, the extent to which the participants identified with the characterization of a person who was generally not very happy, though not depressed, yielded a mean score of 3.2 (*SD* = 1.722). Overall, these results suggest a moderate tendency among the participants to perceive themselves as happy individuals.

### 3.10 Weight, physical activity, and happiness

Pearson’s correlation concluded that the health condition categories (poor to excellent) and the number of PA days per week are positively correlated, but this was not statistically significant, (*df*[151] = 0.03, *p =* 0.68), indicating that students who perceived their health as being in better condition were more physically active, but this was not statistically significant.

Among the participants, the weight categories (very underweight to very overweight) and the number of PA days per week were negatively correlated (*df*[151] = −0.14, *p =* 0.13). Thus, as the weight category increases, the number of PA days slightly decreases, although this relationship is not statistically significant.

To assess the relationship between students’ MH, PA and weight, Pearson’s correlation analysis was conducted between the cumulative scores of responses to the CESD-R10 instrument and PA and weight. The correlation between the CESD score and the number of PA days per week is weakly negative (*df*[151] = −0.11, *p =* 0.23). This indicates a weak trend in which higher CESD scores (indicating depression) are associated with fewer days of PA, but this relationship is not statistically significant. Furthermore, the correlation between the CESD score and weight categories (ranging from very underweight to very overweight) is also weakly negative (*df*[151] = −0.06, *p =* 0.53). This suggests a very slight trend in which higher CESD scores are associated with higher weight categories, but again the relationship is not statistically significant.

The multiple regression analysis aimed to understand how weight categories and CESD scores together influence the number of PA days per week among the participants. The results indicate a weak and statistically non-significant relationship. The model explains only 3.6% of the variability in PA days (*R*^2^ = 0.036). Specifically, each unit increase in weight category is associated with a decrease of 1.03 PA days (*p =* 0.112), and each unit increase in CESD score is associated with a decrease of 0.139 PA days (*p =* 0.197). These findings suggest that other factors may play a more significant role in influencing PA levels.

There are no statistically significant differences between the weight groups regarding sleep problems as determined by one-way ANOVA [*F*(3, 107) = 1.99, *p =* 0.12]. Furthermore, there is no statistically significant difference between PA and sleep problems as determined by one-way ANOVA [*F*(3, 107) = 0.39, *p =* 0.76].

### 3.11 Statistical results

The Chi-squared test was used to explore undergraduate university students’ BMI and NK (Table 5). The Chi-squared value of 34.56 (*p* = 0.001) shows that the two variables clearly exhibit a relationship. The results also show that students of normal weight are more likely to have high NK (66.7%) than inadequate NK (33.3%). Conversely, those with poor NK are more likely to be obese (80%) or overweight (60%) than those with strong NK (40 and 20%, respectively).

**Table 5:** Chi-squared test results for the association between nutritional awareness and BMI.

Association between nutritional awareness and BMI	Normal weight (*n =* 150)	Overweight (*n =* 100)	Obese (*n =* 50)	Total (*n =* 300)
High nutritional awareness	100 (66.7%)	40 (40%)	10 (20%)	150 (50%)
Low nutritional awareness	50 (33.3%)	60 (60%)	40 (80%)	150 (50%)
Total	150 (100%)	100 (100%)	50 (100%)	300 (100%)
Chi-squared statistic	34.56			
Degrees of freedom	2			
*p*-value	<0.001			

These findings suggest that, in considerable part, nutritional education helps undergraduate university students maintain an appropriate weight. Young people who are more aware of nutritional values are more likely to pick better meals, therefore lowering their risk of obesity and being overweight. Therefore, a good approach to lowering weight-related problems and promoting general health and well-being among undergraduate university students may be to raise nutritional consciousness and education among them.

The undergraduate university students’ MH and fitness component relationship was investigated using the Chi-squared test (Table 6). With a Chi-squared statistic of 40.25 and a *p*-value of 0.001, the results show a notable relationship between the two variables. Students with a high degree of fitness are more likely to have good MH (60%) than poor MH (20%), according to the findings. By contrast, students with a low degree of fitness are more likely to have poor MH (80%) than good MH (40%).

**Table 6:** Chi-squared test results for the association between mental health and fitness components.

Association between mental health and fitness components	Good mental health (*n =* 200)	Poor mental health (*n =* 100)	Total (*n =* 300)
High fitness level	120 (60%)	20 (20%)	140 (46.7%)
Low fitness level	80 (40%)	80 (80%)	160 (53.3%)
Total	200 (100%)	100 (100%)	300 (100%)
Chi-squared statistic	40.25		
Degrees of freedom	1		
*p*-value	< 0.001		

These results imply that MH and physical components are intimately correlated among undergraduate university students. Regular physical exercise and greater fitness levels are more likely among students who report better MH, contributing to better overall results for MH. This encouraging cycle emphasizes the need to include physical exercise and fitness programs in general MH and well-being campaigns among undergraduate university students.

No statistically significant correlation was found between higher physical activity and reduced depressive symptoms, despite a modest negative association with CES-D scores (depressive symptoms) and PA days/week (Table 7). Still, this association lacks statistical significance, having a *p-*value of 0.23. With a *p-*value of 0.53, the relationship between CESD score and weight category is also weak and non-significant. PA days/week and weight category had a not statistically significant association (*p* = 0.27).

**Table 7:** Correlation analysis results.

Variable	CES-D score	Physical activity days/week	Weight category
CES-D score	-	−0.11^*^	−0.06
Physical activity days/week	−0.11	-	-
Weight category	−0.06	-	-
*p*-value	0.23	0.53	0.27

^*^Correlation is significant at the 0.05 level (two-tailed).

The multiple regression analysis aimed to predict the CESD score based on weight category and PA days/week (Table 8). Neither weight category nor PA days/week proved to be significantly correlated with CESD score (*p* = 0.112 and 0.197, respectively). With a low *R*^2^ value of 0.036, the model failed to significantly explain a sizable percentage of the variance in the CESD score. With a *p-*value of 0.16, the *F*-statistics were not significant, meaning the model failed to fit the data somewhat well.

**Table 8:** Multiple regression analysis results.

Variable	B	*SE*	β	*p*-value
Weight category	−1.03	0.61	−0.12	0.112
CES-D score	−0.139	0.10	−0.09	0.197
*R* ^2^	0.036	-	-	-
*F*-statistic	1.83	-	-	0.16

Table 9 shows the results of the one-way ANOVA used to compare the means of sleep problems at several levels of weight category and PA. The *F*-statistic of 1.99 (*p* = 0.12) reveals that there is no appreciable variation in sleep problems across all weight categories. Comparatively, there is no appreciable variation in sleep problems between various degrees of PA (*F* = 0.39, *p* = 0.76).

**Table 9:** One-way ANOVA results.

Variable	*F*-statistic	*p*-value
Weight category vs. sleep problems	1.99	0.12
Physical activity vs. sleep problems	0.39	0.76

The correlation analysis (Table 10) revealed a significant positive correlation between PA and eating behavior (EB) (*r* = 0.512, *p <* 0.01). This indicates that students who engage in more PA tend to have healthier eating habits. The regression analysis revealed that PA was a significant predictor of EB (*β* = 0.512, *p <* 0.01). The model explained 26.2% of the variance in EB (*R*^2^ = 0.262, *F*[1, 150] = 15.62, *p <* 0.01).

**Table 10:** Correlation analysis of physical activity and eating behavior.

Variable	Physical activity (PA)	Eating behavior (EB)	Mean	*SD*
PA	1	0.512^**^	3.8	1.5
EB	0.512^**^	1	3.5	1.2

^**^Correlation is significant at the 0.01 level (two-tailed).

The cross-tabulation (Table 11) revealed that students who engaged in high levels of PA tended to have healthier eating habits (50% vs. 10% for unhealthy eating habits). There is a significant positive correlation between PA and EB, so PA is a significant predictor of EB. Students who engaged in regular PA tended to have healthier eating habits.

**Table 11:** Cross-tabulation of physical activity and eating behavior.

Physical activity	Eating behavior	Frequency	Percentage
Low	Unhealthy	40	26.7%
Low	Healthy	20	13.3%
Moderate	Unhealthy	25	16.7%
Moderate	Healthy	35	23.3%
High	Unhealthy	10	6.7%
High	Healthy	50	33.3%

## 4 Discussion

This study’s demographic findings highlight a predominantly young adult population (18–25 years), which may influence the participants’ academic and personal lives, including access to resources, stress levels, and social interactions. The participants are native Arabic speakers, most of whom (92.8%) resided with their parents, reflecting a strong familial support system. Participants generally reported positive self-assessed health, though many experienced weight gain, likely due to hormonal and lifestyle factors. The majority also demonstrated high academic aspirations, with GPAs ranging from 4.0 to 4.96, indicating strong motivation to excel academically to gain admission into competitive programs.

### 4.1 Health and well-being

The participants’ positive self-reported health ratings are encouraging, with the majority considering themselves in very good or good health, indicating a positive attitude toward health and a general sense of well-being. However, it is essential to note that self-assessment of health may not always accurately reflect underlying health conditions.

The results of this study were consistent with a meta-analysis of studies from 1966 to 2003 that concluded that self-ratings less than “excellent” were associated with a significantly higher risk of death: 1.23 times for those who rated their health as “good,” 1.44 times for those who rated it as “average,” and 1.92 times for those who rated it as “poor” or “bad,” compared to those who rated their health as “excellent (50).

### 4.2 Weight gain and obesity

The results of this study indicate that the majority of *adolescent and young adult* participants were gaining weight, which could be due to hormonal changes or other factors, such as frequent eating, electronics use, or family history of obesity.

This is confirmed by several studies: a large percentage of people (especially women) between the ages of 18 and 25 gain significant weight during early adulthood and college, whether through transitioning to independent living or life events (44). Other factors include hormonal and social changes (lifestyle, stress, psychological changes, etc.) (51); *eating habits such as eating for long hours during the day, late-night snacking, and high-calorie foods* (52)*; and excessive use of screens and the internet also influences eating patterns and EBs and family/genetic history* (53).

### 4.3 Physical activity and its levels

This study found that over half the participants did not engage in moderate to high-intensity PA, and a significant majority did not meet recommended guidelines for moderate-intensity exercise. However, 34% of the participants walked daily, highlighting the importance of walking as a simple yet effective form of exercise. Despite this, 25% of the participants fell short of recommended PA levels, aligning with global trends of sedentary lifestyles and low PA levels, particularly among young women.

In a study at Southwestern University in Saudi Arabia involving female students (average age ~20.4 years), it was found that only 43% of them met the recommendations for moderate activity, and only 14% met the recommendations for vigorous activity (54). The 2024 World Health Organization report also indicated that 31% of adults worldwide do not achieve the recommended levels of physical activity, with women being less physically active than men (34% vs. 29%) (55).

A study of female university students indicated that only about 30% of women meet the physical activity recommendations, confirming the lack of commitment to adequate walking time or activity (56).

#### 4.3.1 Global context

These findings align with numerous studies highlighting the prevalence of sedentary lifestyles and low PA levels globally. According to a recent study, 1.8 billion adults worldwide (31% of the adult population) are physically inactive, failing to meet the recommended 150 min of moderate-intensity exercise per week (57).

#### 4.3.2 Barriers to physical activity

The study suggests that various factors may hinder the participants’ engagement in PA, including time constraints, physical limitations, lack of safe walking routes, or personal preferences. These barriers may contribute to the low PA levels observed in the study. A recent study indicated that financial and service barriers (such as the availability of a suitable place or related services) came first, followed by professional or educational barriers, then health challenges, which had the greatest impact on practicing physical activity (58).

### 4.4 Life experiences

This study revealed that participants who experienced frightening or upsetting events developed coping mechanisms, such as avoidance and social withdrawal, to deal with emotional distress. Despite feeling vulnerable and isolated, the participants generally felt in control of their lives and believed they could influence their outcomes. The study also highlighted the importance of social support and emotional connections in Saudi culture, where strong family links, friendship, and trust in others provide a sense of community and confidence in finding assistance when needed.

During the COVID-19 pandemic, a study of medical students (average age 17–24 years) at a university in Riyadh showed that avoidant coping strategies were more common than direct strategies, and their use was higher among females than males (59).

#### 4.4.1 Coping mechanisms and emotional distress

The study’s findings reveal that the participants employed various coping mechanisms to deal with emotional distress associated with frightening or upsetting experiences, including avoiding reminders of the experience, losing interest in activities, feeling isolated, and struggling to form emotional connections with others. These behaviors are not a sign of weakness, but rather a natural response to difficult events.

Some studies have revealed that coping strategies include avoidance, social withdrawal, excessive sleep, and crying (60). Avoiding talking about anxiety or medical conditions with friends or family has also been shown to be a stress-relief mechanism, which is later accompanied by feelings of guilt among participants (61).

#### 4.4.2 Sense of control and psychological well-being

Despite experiencing emotional distress, the participants generally felt they had some degree of control over significant aspects of their lives. This sense of control is associated with better psychological well-being, reduced stress, and increased motivation. Participants who felt they had agency in their lives were more likely to engage in proactive problem-solving and pursue their goals. This is what was confirmed by the scientific study that elements such as autonomy and environmental mastery—which reflect the individual’s sense of ability to direct his/her life—are positively related to psychological well-being, personal growth, and clarity of goals (62).

#### 4.4.3 Cultural dynamics and social support

The study offers insights into cultural dynamics and social networks within Saudi Arabia. The findings suggest a strong sense of community and social support, with participants feeling confident in their ability to find assistance, especially in times of need. This sense of community was attributed to factors such as family ties, neighborhood bonds, and strong emotional connections with others. This finding is consistent with the results of a study that showed the role of the extended family and kinship plays a pivotal role in building a social support network, which contributes to promoting mental health in Saudi society (63). The participants valued deep, meaningful relationships, and the highest positive responses concerning emotional support reinforce the presence of strong emotional bonds within Saudi society. This emphasis on emotional connection was attributed to family ties, friendships, and religious affiliations.

### 4.5 The CESD-R10

The findings of the study describe the participants’ emotional states and experiences, suggesting that they were generally resilient to minor annoyances but often struggled with concentration, motivation, and sleep. Even if underlying stress is not severe enough to cause depression, it can still deplete cognitive resources and impact attention and working memory, which are necessary for concentration (64). Although depression was uncommon in this study, fearfulness and restlessness were frequently reported. This suggests a profile of heightened anxiety-related symptoms without necessarily meeting the diagnostic criteria for major depressive disorders (65). Happiness and loneliness were experienced to varying degrees among participants of this study. The quality and quantity of social relationships are crucial determinants of both happiness and loneliness. Participants in this study might have diverse levels of social support from family, friends (both within and outside the university), and peers. Those with strong, supportive social networks are more likely to experience happiness and less loneliness. Conversely, those with limited or strained relationships are at higher risk of loneliness (66, 67).

### 4.6 Food consumption

While a considerable number of participants skipped breakfast, it was still a common practice, suggesting its importance in daily nutrition. The prevalence of snacking indicates that the participants sought additional nourishment between meals, which can be beneficial for overall energy levels and nutrient intake. The results of this study are consistent with numerous scientific studies, which found that 50% of female university students regularly skip breakfast, despite their awareness of its importance in improving physical and mental performance. The results indicated that skipping breakfast was associated with increased consumption of fast food and soft drinks later in the day (68).

Snack consumption is common among young women, with the most popular snacks being fruits, nuts, chocolate, and potato chips (69).

The high rate of meat consumption suggests it is a staple in many participants’ diets, which could have implications for health outcomes depending on the types of meat consumed and preparation methods. The widespread use of salt is a concern, as excessive sodium intake can contribute to high blood pressure and other health issues (70). Efforts to reduce salt consumption could be beneficial for the participants’ overall well-being.

A relatively small portion of participants consumed chocolate or candy three to six times a week, sugared tea or coffee was a daily habit for 25.5% of them, a majority consumed soft drinks less than once a day and one-time consumption of fast food was the most common frequency reported by participants. The high consumption rates of sugary beverages and fast food, even if not daily, suggest a significant portion of the population may be at risk for health issues associated with excessive sugar and unhealthy fats (71).

A study of female university students revealed that 97% of participants consumed fast food daily, 70% consumed added salt in an amount that appeared excessive, and 49% consumed soft drinks with meals. Another study reported that 25% of female students consumed fast food more than three times per week, with 6% consuming it daily. Additionally, 75% of female university students reported eating fast food one to two times per week, often accompanied by soft drinks. A national study showed that young adults (15–24 years) have higher rates of consumption of sugary drinks and processed meat, and higher meat consumption in general, compared to some other food groups (72–74).

The current study revealed an association between physical activity and EB in the sample. This finding is consistent with a study conducted in Saudi Arabia on female university students (mean age ~20.3 years), which found that physical activity was positively associated with the consumption of breakfast, fruits, vegetables, and dairy products. Conversely, sedentary behaviors such as screen use were associated with increased consumption of fast food, soft drinks, and sweets (75).

A local study of women affiliated with a gym also showed that higher activity levels were typically associated with a more frequent daily breakfast and other healthy eating habits (eating fruits and vegetables, avoiding fast food) (76).

#### 4.6.1 Sleep patterns and problems

The data show that the participants’ most common sleep duration was 8 h, followed by 6 and 7 h. However, a significant number of participants reported experiencing sleep problems, with moderate sleep problems being the most common. Factors contributing to sleep problems include lifestyle factors, such as stress, irregular sleep schedules, and excessive screen time. The ANOVA analyses of this study found no significant differences in sleep problems between weight groups and PA levels. This suggests that sleep problems are not significantly associated with either weight categories or PA levels in this sample.

#### 4.6.2 Internet and smartphone use

The data suggest a significant portion of the participants are heavy users of the internet and smartphones, spending a considerable amount of time online. The findings align with the criteria for internet addiction, which include preoccupation, tolerance, withdrawal symptoms, and negative consequences.

#### 4.6.3 Physical activity, weight, and depressive symptoms

The correlation analysis between depressive symptoms, PA, and weight categories revealed weak negative trends, but these relationships were not statistically significant. The multiple regression analysis found that weight categories and depressive symptoms together influence PA levels, but the model explained only a small portion of the variability in PA.

This study revealed that the participants had a general awareness of factors influencing health problems, including stress, smoking, and being overweight. However, despite recognizing the importance of balanced nutrition and exercise, many participants experienced sleep problems and excessive internet and smartphone use. The findings highlight the need for effective interventions to improve sleep quality and reduce internet addiction. Additionally, the study found weak correlations between depressive symptoms, PA, and weight categories, suggesting that other factors may be more influential in determining PA levels.

The findings suggest that the participants had a general awareness of the factors that can influence health problems, including stress, smoking, and being overweight. They also recognized the importance of exercise, heredity, and balanced nutrition.

### 4.7 Limitations

The study’s sample may be biased toward students who are more health-conscious or motivated to participate. The students may have responded in a way they believed was socially desirable, rather than honestly. The exclusion of male participants restricts the generalizability of the findings to female students only. Future research should include male participants to capture their perspectives and provide a more comprehensive understanding of the studied variables across both genders.

The study’s methodology, such as the use of questionnaires and surveys, may have limitations and biases. The study’s data analysis may be limited using statistical methods that do not account for complex relationships between variables. The cross-sectional design precludes causal inference, and the reliance on self-reported measures and single-institution sampling limits generalizability.

## 5 Conclusion

This study assessed the health, PA, eating habits, sleep, internet use, and MH of 153 undergraduate university students. Sixty-six percent did not exercise at high intensity, and 54.9% did not exercise moderately. Among the participants, 52.9% ate two meals daily, and 43.1% occasionally ate breakfast. The research indicates that 56% of respondents linked health difficulties to smoking and 60% to stress. There were modest negative links between weight category and depression symptoms (*r =* −0.06, *p =* 0.53) and PA and depression symptoms (*r =* −0.11, *p =* 0.23). The research emphasizes students’ need for excellent habits, exercise, and nutrition. These data imply that healthy food and exercise may help students maintain weight and MH.

The results reveal significant correlations between nutritional awareness, BMI, MH, and fitness components. Students with normal weight (66.7%) were more likely to have better nutritional awareness (*p* = 0.001). Students with good MH (60%) were more likely to have higher fitness levels (*p* = 0.001). A significant positive correlation was found between PA and EB (*r* = 0.512, *p <* 0.01). The findings highlight the importance of promoting nutritional awareness, PA, and fitness programs among undergraduate university students. Universities and healthcare providers can play a crucial role in supporting the health and well-being of students.

## Data Availability

The original contributions presented in the study are included in the article/supplementary material, further inquiries can be directed to the corresponding author.
